# The complete mitochondrial genome of the oceanic squid: *Chiroteuthis picteti* (Oegopsida, Chiroteuthidae)

**DOI:** 10.1080/23802359.2018.1437831

**Published:** 2018-02-12

**Authors:** Hana Kim, Cheol Yu, Hyung June Kim, Dong Won Kang, Yun-Hwan Jung

**Affiliations:** a Department of Taxonomy and Systematics, National Marine Biodiversity Institute of Korea, Janghang-eup, Republic of Korea;; b Department of Biological Sciences, Inha University, Incheon, Republic of Korea

**Keywords:** Mitochondrial genome, *Chiroteuthis picteti*, Chiroteuthidae, Mollusca

## Abstract

The mitogenome sequence of *Chiroteuthis picteti* Joubin, 1894 (Oegopsida, Chiroteuthidae), a species of Oceanic squid, was first determined in the genus *Chiroteuthis* as well as in the family Chiroteuthidae. The circular genome of *C. picteti* is 20,851bp in size, has six duplicated genes (*cox1*, *cox2*, *cox3*, *atp6*, *atp8*, and tRNA^Asp^). The mitogenome of *C. picteti* has high A + T content (71.6%) similar to other Oegopsid (A 38.2%; C 18.3%; G 10.1%; T 33.4%). The mitogenome of *C. picteti*, the first reported of its genus, will be useful for inferring the phylogenetic relationships among the members of Chiroteuthidae, within the Oegopsids.

The genus *Chiroteuthis* d’Orbigny, 1841 contains only nine species which is widely distributed throughout the world’s oceans, known from the subarctic, temperate, tropical, and the subantarctic waters (Roper and Young 1998). *Chiroteuthis picteti,* called an oceanic squid, is rare species in the Korean ocean because it lives deep in the sea of tropical and subtropical areas. In this study, we determined the complete mitochondrial genome of *C. picteti,* first in the genus *Chiroteuthis*.

Specimens of *C. picteti,* collected from a fixed shore net of subtidal zone, in Yeongdeok-gun of east sea of Korea. The voucher specimen was deposited in National Marine Biodiversity Institute of Korea (MABIK MO00171532). The genomic DNA was extracted from the muscle tissue and the mitogenome sequences were analyzed in two ways: First, three contigs of long-length were obtained using Illumina Hiseq2000 sequencing platform (Macrogen, Seoul, Korea); Then, two gap sequences of 600 bp and 823 bp were amplified through the PCR with primer sets designed from the sequence obtained by the first method, and sequenced by the Sanger method. These sequences were assembled and annotated in comparison with mitogenome sequences of species belonging to the order Oegopsida previously reported by Yokobori et al. (2004) using Geneious 9.1.8 (Kearse et al. 2012). Additionally, we used the mitochondrial genome annotation (MITOS) server (Bernt et al. 2013) and tRNAscan-SE server (Lowe and Chan 2016) for annotation. A phylogenetic tree was constructed using MEGA6 (Tamura et al. 2013).

The circular mitogenome of *C. picteti* (GeneBank accession number MG833837) is 20,851 bp in length and *Chiroteuthis* mitochondrial genes have six duplicated genes (*cox1*, *cox2*, *cox3*, *atp6*, *atp8*, and tRNA^Asp^) that are features of Oegopsids mitogenome (Kawashima et al. 2013). One amino-acid change (A to G) was identified in the duplicated *COX3* gene, and six amino-acid changes (F to M, M to I, D to Q, N to I, A to G, and N to Y) were identified in the duplicated *ATP6* gene. The mitogenome of *C. picteti* has a high A + T content (71.6%) similar to the other Oegopsids (A. 38.2%; C 18.3%; G 10.1%; T 33.4%). All PCGs get off by the typical ATG start codon. Seven PCGs (*cox3*, *nad3*, *cox1*, *atp8*, *nad5*, *nad6*, *nad1*) use TAA for the stop codon and five (*cox2*, *atp6*, *nad4*, *nad4l*, *cytb*) ends with TAG while one (*nad2*) genes have an incomplete stop codon, T––. The lengths of tRNA genes range from 63 to 74 bp and all tRNAs have the typical clover leaf structure.

To confirm the molecular phylogenetic position of *C. picteti*, we conducted the Neighbour-joining analysis with the K2P model in MEGA6 and dataset utilizing concatenated sequences of 13 PCGs except on one side of the duplicated genes (*cox1*, *cox2*, *cox3*, *atp6*, *atp8*). *C. picteti* is closely related to *Architeuthis dux* and order Oegopsida including *Chiroteuthis* is a monophyletic group which is strong support for prior studies (Kawashima et al. 2013) (Figure 1). The mitogenome of *C. picteti*, the first report of its genus, will be useful for inferring the phylogenetic relationships among the members of Chiroteuthidae within the Oegopsids.

**Figure 1.**
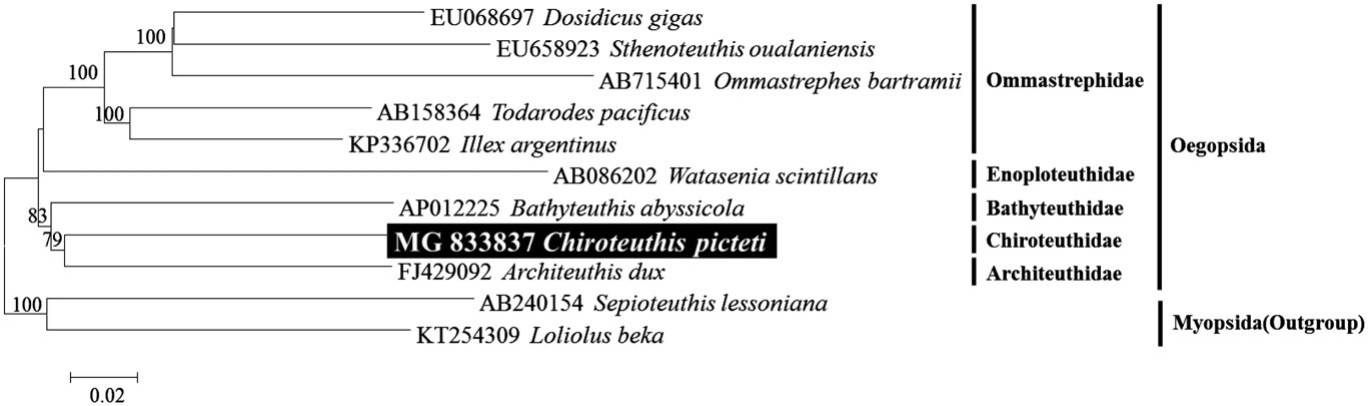
Neighbour-joining (NJ) tree based on the mitogenome sequences of *C. picteti* with eight other related species in Oegopsid. *Sepioteuthis lessoniana* and *Loliolus beka* derived from Myopsid was used as an outgroup for tree rooting. Numbers above the branches indicate NJ bootstrap values from 1000 replications.
